# Super-resolution image projection over an extended depth of field using a diffractive decoder

**DOI:** 10.1038/s41377-026-02320-7

**Published:** 2026-05-18

**Authors:** Hanlong Chen, Çağatay Işıl, Che-Yung Shen, Shiqi Chen, Tianyi Gan, Mona Jarrahi, Aydogan Ozcan

**Affiliations:** 1 Electrical and Computer Engineering Department, University of California, Los Angeles, CA USA; 2 Bioengineering Department, University of California, Los Angeles, CA USA; 3 California NanoSystems Institute (CNSI), University of California, Los Angeles, CA USA

**Keywords:** Applied optics, Terahertz optics

## Abstract

Image projection systems must be efficient in data storage, computation and transmission while maintaining a large space-bandwidth-product (SBP) at their output. Here, we introduce a hybrid image projection system that achieves extended depth-of-field (DOF) with improved resolution, combining a convolutional neural network (CNN)-based digital encoder with an all-optical diffractive decoder. A CNN-based encoder compresses input images into compact phase representations, which are subsequently displayed by a low-resolution (LR) projector and processed by an analog diffractive decoder for all-optical image reconstruction. This optical decoder is completely passive, designed to synthesize pixel super-resolved image projections that feature an extended DOF while eliminating the need for additional power consumption for super-resolved image reconstruction. Our pixel super-resolution (PSR) image projection system demonstrates high-fidelity image synthesis over an extended DOF of ≥250*λ*, where *λ* is the illumination wavelength, concurrently offering up to ~16-fold SBP improvement at each lateral plane. The feasibility of this approach is validated through proof-of-concept experiments conducted in both the terahertz and visible spectral regions, demonstrating the scalability of our hybrid system across different wavelengths. This image projection architecture can reduce data storage and transmission requirements for display systems without imposing additional power constraints on the optical decoder. Beyond extended DOF PSR image projection, the underlying principles of this approach can be extended to various applications, including optical metrology and microscopy.

## Introduction

Augmented and virtual reality (AR/VR) systems hold immense promise across diverse fields, including education, entertainment, and healthcare^1,2^. These systems aim to deliver immersive user experiences. Near-eye displays, while crucial for achieving this objective, have presented a hurdle in realizing the full potential of AR/VR technology. A critical issue plaguing most near-eye displays, including stereoscopic, autostereoscopic, and multi-view systems, is the well-documented discomfort and fatigue experienced by users^3,4^. This primarily stems from the mismatch between vergence and accommodation cues of the eye. Holographic displays, in contrast to these widely adopted approaches, possess the ability to offer all depth cues of the human visual system. This capability can potentially alleviate eye strain and visual discomfort associated with vergence-accommodation conflict^4–9^, which is important for AR/VR systems. However, the space-bandwidth product (SBP) of holographic displays is constrained by the limited pixel count of spatial light modulators (SLMs). In addition to this, further research into advanced computer-generated holography (CGH) techniques and supporting hardware is crucial for enabling real-time, full-parallax holographic displays with low power consumption^7,8,10,11^. Advances in machine learning techniques have also been applied to improve hologram calculation in terms of accuracy and speed^12–21^, as well as to enhance the SBP of SLMs^22,23^ through passive surfaces. An alternative strategy involves computing holograms on a remote server and transmitting them to local receivers with limited, low-cost processors^4^. However, hologram compression presents challenges, as conventional image compression techniques like JPEG (Joint Photographic Experts Group) inevitably sacrifice the fine details critical for synthesizing high-quality holographic images. These limitations highlight the need for innovative image projection approaches that can simultaneously address the current challenges associated with data transmission, processing power, and restricted SBP in display technologies.

Here, we present a deep learning-enabled image projection approach that synergistically integrates a digital encoder with a passive all-optical decoder, achieving both extended depth of field (DOF) and pixel super-resolution (PSR). This hybrid architecture employs a convolutional neural network (CNN) to efficiently encode high-resolution image information into compact phase-only representations, which are subsequently displayed by a low-resolution (LR) projector and processed by a passive diffractive decoder to synthesize super-resolved images over an extended DOF. We validated the scalability of this framework through comprehensive numerical simulations and proof-of-concept experiments conducted in both the terahertz and visible parts of the spectrum, demonstrating its ability to project images over a DOF of $\ge 250{\rm{\lambda }}$ with approximately 16-fold SBP improvement compared to the SBP of the input projector, where $\lambda$ is the illumination wavelength. Furthermore, we rigorously evaluated the system’s external generalization capabilities on structurally distinct unseen object classes and its robustness against non-idealities, including component misalignment, phase bit depth, and quantization. This digital encoder–analog decoder architecture provides a viable path to address data storage and transmission requirements while reducing computational costs, as it incurs no additional power consumption or significant latency at the user end, leveraging the intrinsic advantages of its all-optical passive decoder. This hybrid super-resolution image projection system, with its extended DOF and adaptability across different wavelengths, might find various applications in optical metrology and microscopy, as well as in next-generation holographic displays.

## Results

**Fig. 1:**
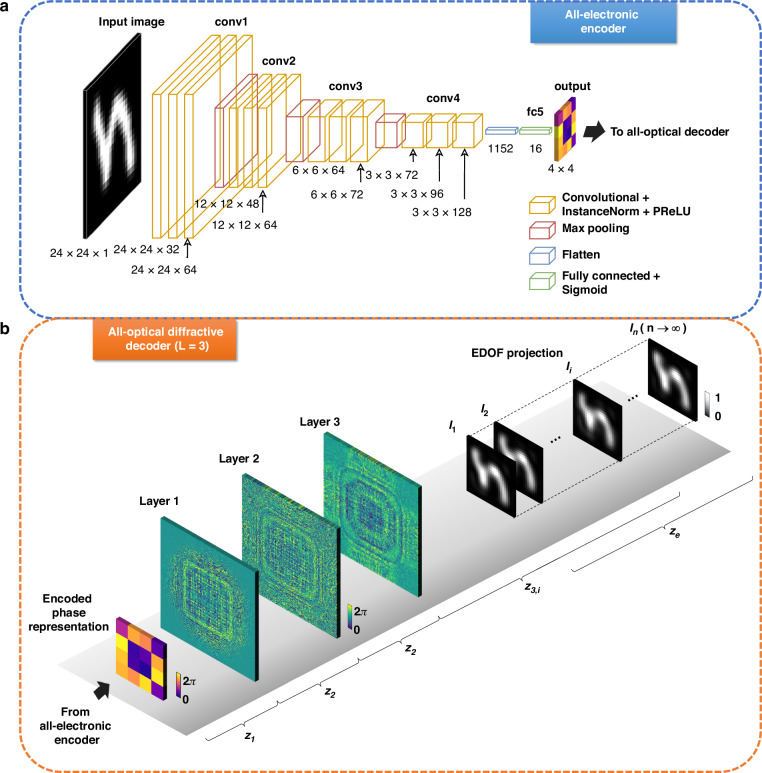
Schematic illustration of the hybrid PSR image projection system with a digital encoder and a diffractive optical decoder. **a** The digital encoder transforms grayscale input images (each with $24\,\times 24$ pixels) into compact phase-encoded representations of size $\frac{24}{k}\times \frac{24}{k}$ pixels, where $k=6$ is shown here as an example. **b** The diffractive decoder all-optically decodes and modulates the wavefront to achieve a continuous projection with an extended depth of field, EDOF (${z}_{e}$). The distance ${z}_{1}$ represents the axial distance from the input plane to the first diffractive layer; ${z}_{2}$ denotes the axial spacing between adjacent passive diffractive layers. ${z}_{\mathrm{3,1}}$ and ${z}_{3,n}$ indicate the axial distances from the last diffractive layer to the nearest and farthest projection planes, respectively. For demonstration, discrete positions within this *continuous* projection field are shown, denoted as ${z}_{\mathrm{3,1}},{z}_{\mathrm{3,2}},\ldots ,{z}_{3,i},\ldots ,{z}_{3,n}$, with the corresponding cross-sectional images ${I}_{1},{I}_{2},\ldots ,{I}_{i},\ldots ,{I}_{n}$, respectively

The hybrid architecture of our extended DOF PSR image projection system integrates a digital encoder with an all-optical passive diffractive decoder, as depicted in Fig. 1. The electronic encoder, shown in Fig. 1a, employs a lightweight CNN-based encoder to process the input image through a sequence of convolutional layers (Fig. 1a). Each layer is equipped with instance normalization^24^ and Parametric Rectified Linear Unit (PReLU) activation^25^. In our proof of concept demonstration, this CNN-based encoder is designed to compress grayscale images with $24\times 24$ pixels into compact phase representations with $\frac{24}{k}\times \frac{24}{k}$ pixels where $k=6$ in Fig. 1a – i.e., a reduction of 36-fold in the total number of pixels per image. The all-optical analog decoder is composed of three diffractive layers ($L=3$), illustrated in Fig. 1b, and it is jointly designed/optimized to process the encoded compact phase patterns to synthesize high-resolution images over an extended DOF (EDOF), denoted by ${z}_{e}$. Stated differently, our projection framework performs a continuous EDOF projection while improving the effective SBP of the reconstructed images, which is illustrated using 2D projection slices at the selected discrete positions represented by ${z}_{\mathrm{3,1}},{z}_{\mathrm{3,2}},\ldots ,{z}_{3,i},\ldots ,{z}_{3,n}$, with the corresponding cross-sectional images denoted as ${I}_{1},{I}_{2},\ldots ,{I}_{i},\ldots ,{I}_{n}$, respectively. To train our hybrid models, we utilized the EMNIST^26^ handwritten letter dataset, comprising 80,000 training, 8800 validation, and 14,800 testing images, each with $24\times 24$ pixels, and employed the AdamW optimizer, as detailed in the **Methods** section.

**Fig. 2:**
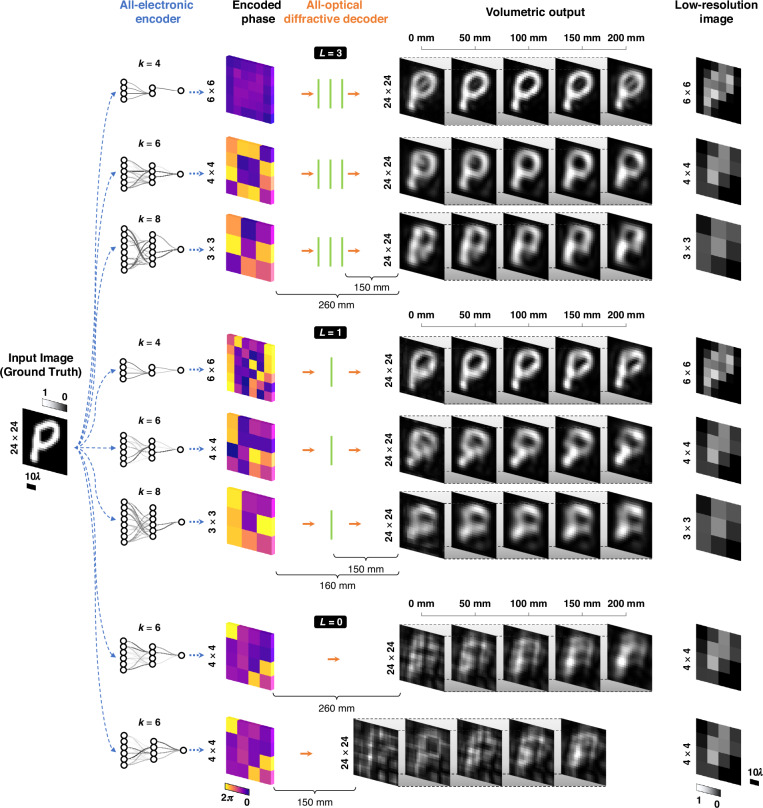
Performance comparison of hybrid PSR image projection systems. The input images (each $24\times 24$ pixels) are processed through our PSR image projection systems trained for varying PSR factors ($k=4,\,6,\,8$) and different numbers of passive diffractive layers ($L\,=\,1,\,3$) for the diffractive decoder. Each row represents a distinct system configuration with jointly trained encoder-decoder pairs, optimized independently from other configurations. The outputs are continuous projections with an extended DOF, i.e., ${z}_{e}=200\,{\rm{mm}}\,( \sim 266.85{\rm{\lambda }})$. For illustration purposes, several propagation distances, i.e., 0 mm, 50 mm (~66.71λ), 100 mm (~133.43λ), 150 mm (~200.14λ), 200 mm (~266.85λ), are selected to showcase cross-sectional views. The rightmost column displays the low-resolution images corresponding to each PSR factor, $k$. The bottom two rows illustrate system configurations with *no* diffractive layers (the encoder-only cases, where $L\,=0$), serving as a comparison, highlighting the significance of the diffractive decoder

Figure 2 compares the image projection performances of diffractive projection systems with different numbers of passive diffractive layers (*L* = 1, 3) trained for different PSR factors (k = 4, 6, 8). Each configuration, represented by a distinct row in Fig. 2, is separately trained (see Supplementary Figure S1 for the decoders’ phase profiles corresponding to each design), and each design is blindly evaluated using the test dataset not seen by the models during the training phase. As an example, a test image of handwritten “*P*” is processed through the diffractive image projection systems and the resulting EDOF image projections are presented using lateral cross-sections at selected propagation distances, which showcase the system’s ability to maintain high-fidelity projections throughout an extended DOF, axially spanning ${z}_{e}\approx 266.85\lambda$. For instance, the three-layer-based decoder configurations (*L* = 3) effectively maintain a similar image quality across the entire axial depth range at PSR factors of *k* = 4, 6, and 8. On the other hand, single-layer decoder configurations ($L=1$) show a more pronounced trade-off between resolution and DOF performance (see Fig. 2). Notably, configurations with a free-space decoder (*L* = 0, i.e., no diffractive decoder) present significantly deteriorated images synthesized across the same DOF, confirming the essential role of the diffractive decoder layers jointly optimized with the digital encoder at the front-end.

**Fig. 3:**
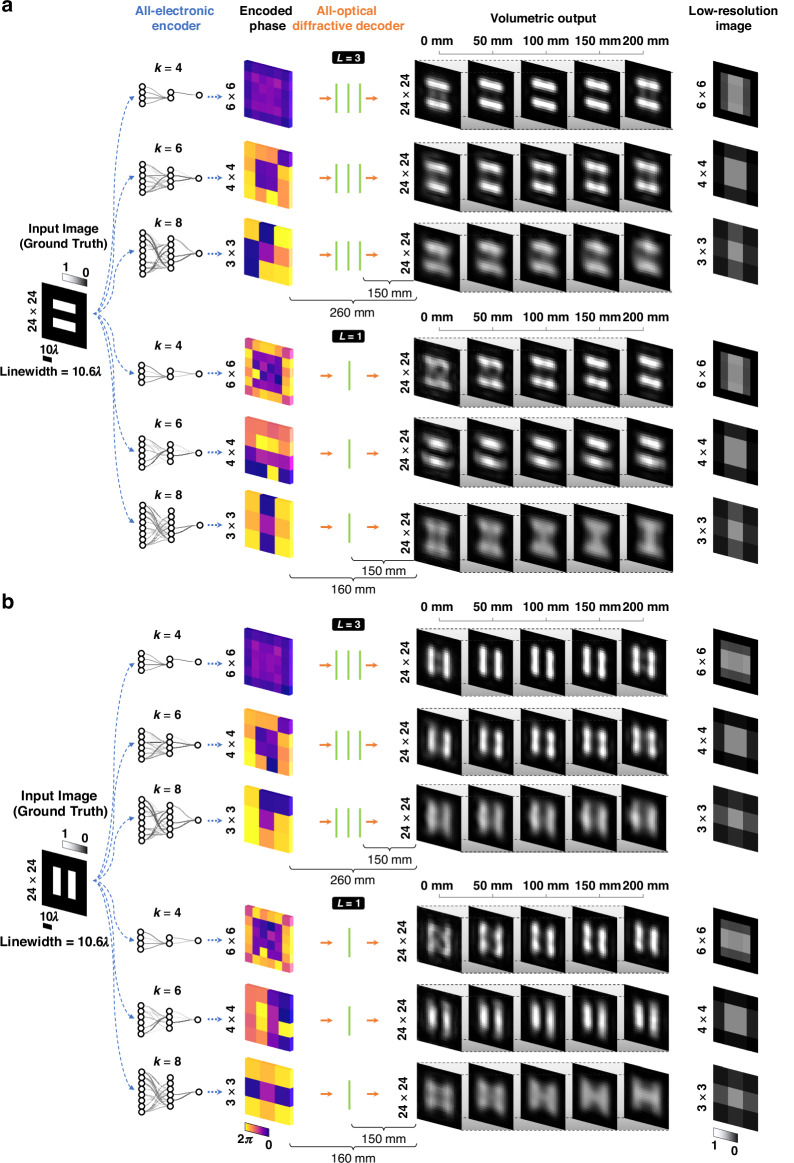
External generalization performance of hybrid PSR image projection systems on grating patterns—never seen before. This figure demonstrates the efficacy of the system in generalizing to unseen data types, utilizing encoder-decoder models trained with the EMNIST dataset for each combination of $k$ and $L$. **a** Horizontal and **b** vertical grating inputs ($24\times 24$ pixels) are processed through each configuration. Encoded phase patterns and EDOF outputs, axially sampled at 0 mm, 50 mm (~66.71λ), 100 mm (~133.43λ), 150 mm (~200.14λ), 200 mm (~266.85λ) showcase continuous projection over a DOF of 200 mm (~266.85λ). The rightmost column presents the corresponding low-resolution images for each PSR factor, $k$

We further explored our hybrid projection system by demonstrating its *external generalization* capabilities in Fig. 3. Horizontal and vertical gratings, which differ from the training and test datasets used in the previous results, were employed to test the external generalization of our extended DOF PSR image projection system. As illustrated in Fig. 3, despite being trained using solely the EMNIST dataset, the three-layer-based decoder models accurately synthesized images of gratings with a linewidth of $10.6\lambda\,( \sim 7.94\,\mathrm{mm})$ over an extended DOF $\{z_3\in{\mathbb{R}} \mid 150\le z_3 \le 350\}$ for different PSR factors of *k* = 4, 6, 8. Notably, the single-layer designs for $k=4$ and 6 also performed image projections over an extended DOF with some minor degradation in image quality compared to three-layer decoder results. Figure 3 further reveals that our extended DOF method achieves up to $\sim 16$-fold SBP improvement compared to the input/encoding plane, in addition to proving external generalization of the presented framework. Specifically, the model trained for $k=8$ successfully projected gratings with a linewidth of $10.6\lambda\,( \sim 7.94\,{\rm{mm}})$, translating to an effective pixel size of $5.3\lambda\,( \sim 3.97\,{\rm{mm}})$ at the output plane. These results are obtained using an effective pixel size of 16 mm ($\sim 21.35\lambda )$ at the encoded phase plane, corresponding to a $\sim 16$-fold improvement in the effective SBP^22,27^. Even smaller linewidths can be imaged through this diffractive platform by specifically training it, end-to-end, for the projection of densely-packed fringe patterns as illustrated in Supplementary Figure S2.

**Fig. 4:**
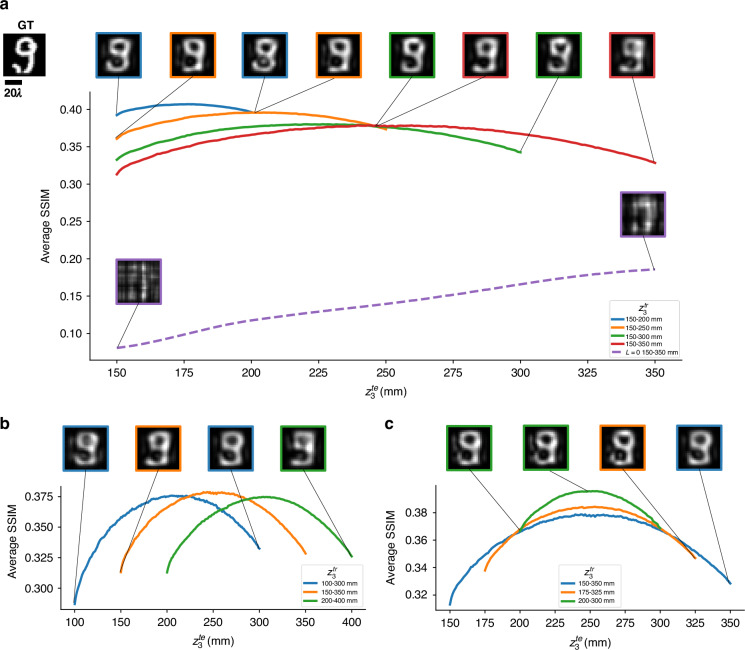
Quantitative analysis of the hybrid PSR image projection systems across varying z_3_ ranges. Each solid curve represents a hybrid system ($L=3$, $k=6$) trained over a specific ${z}_{3}^{{tr}}$ training range and evaluated over the corresponding ${z}_{3}^{{te}}$ testing range, as indicated in the legend. The dashed purple curve depicts the performance of a projection system without a diffractive decoder (i.e., $L=0$), trained and evaluated over an equivalent axial depth range. Representative reconstructed images at selected ${z}_{3}^{{te}}$ positions are displayed above the corresponding curves. **a** Impact of increasing the depth range on the image projection fidelity, with all ranges (${z}_{3}^{{te}}$) starting from 150 mm $( \sim 200.14{\rm{\lambda }})$. **b** Performance comparison for depth ranges of a constant width ${z}_{e}$ of 200 mm $( \sim 266.85{\rm{\lambda }})$ but varying central positions, elucidating the influence of range placement on the PSR image projection quality. **c** Average SSIM as a function of ${z}_{3}^{{te}}$ for axial ranges sharing a common midpoint at 250 mm$\,( \sim 333.56{\rm{\lambda }})$ but differing in width, ${z}_{e}$

Figure 4 further presents quantitative and visual analysis of our hybrid image projection approach across varying projection distances and depths. For this purpose, different three-layer designs ($L=3$) targeting a PSR factor of $k=6$ were trained for various ${z}_{3}^{{tr}}$ ranges and tested using different ${z}_{3}^{{te}}$ ranges. Here, $k=6$ was chosen as it strikes a balance between high compression and reconstruction fidelity, thereby providing a more stringent and representative evaluation of the hybrid diffractive decoder’s performance. Figure 4a elucidates the impact of expanding the depth range ${z}_{e}$ to 50 mm (~66.71λ), 100 mm (~133.43λ), 150 mm (~200.14λ), and 200 mm (~266.85λ), while maintaining a fixed starting point ${z}_{\mathrm{3,1}}=150\,{\rm{mm}}\,( \sim 200.14{\rm{\lambda }})$. As the DOF broadens, the peak average structural similarity index measure (SSIM) gradually declines, indicating a natural trade-off between the DOF and image projection fidelity. In contrast, an image projection system with a free-space-based decoder (i.e., $L=0$, trained and evaluated over an equivalent axial range), shown with the dashed purple curve in Fig. 4a, exhibits significantly degraded performance, once again highlighting the crucial role of the passive diffractive decoder in extending the DOF while maintaining high image projection fidelity. Similarly, Fig. 4b, c illustrate the influence of depth range placement and its axial width on the image projection performance. While narrower DOF ranges yield higher SSIM values at the output projection volume, the system, in general, maintains high image quality and discernible features across all the tested DOF ranges reported in Fig. 4.

To quantitatively characterize the effective DOF enabled by the presented hybrid image projection architecture in the terahertz domain, we further evaluated our design using resolution test targets and image-quality metrics, including Michelson contrast and peak signal-to-noise ratio (PSNR). DOF is commonly defined based on the preservation of image contrast under defocus^28,29^. Accordingly, defining the DOF as the axial range over which an image contrast metric remains above a specified fraction of its peak value (e.g., contrast > 50%) is a widely adopted practical criterion in imaging and projection systems^30,31^. In this work, we adopt a more stringent threshold, requiring the Michelson contrast to remain above 90% of its peak value, which corresponds to minimal perceptual degradation and reliable preservation of fine spatial features. In addition, PSNR is used as a complementary metric to quantify overall image reconstruction fidelity. As shown in Supplementary Fig. S3a, these metrics were analyzed both within and beyond the training axial depth range, enabling a direct assessment of the system’s external generalization capability along the axial direction. Consistent with the SSIM trends discussed above, the results indicate that image contrast and PSNR values remain stable across the trained DOF range and degrade gradually outside this range. Based on these quantitative metrics, using a PSNR threshold of 1 dB drop below the maximum and a Michelson contrast threshold of 90% of the peak value, the effective DOF was determined to span from ${z}_{3}$ = 170.5 mm to ${z}_{3}$ = 357.7 mm, spanning an axial width of $\sim 250{\rm{\lambda }}$, within which the system consistently delivers high-contrast, high-fidelity image projections.

Complementing this quantitative analysis, Supplementary Fig. S3b shows representative projected images at multiple axial locations ${(z}_{3})$, further demonstrating the preserved contrast and resolvable features across a wide span of image projection distances. This consistent visual performance corroborates the quantitative findings and highlights the decoder’s ability to mitigate diffraction-induced degradation over extended depths.

**Fig. 5:**
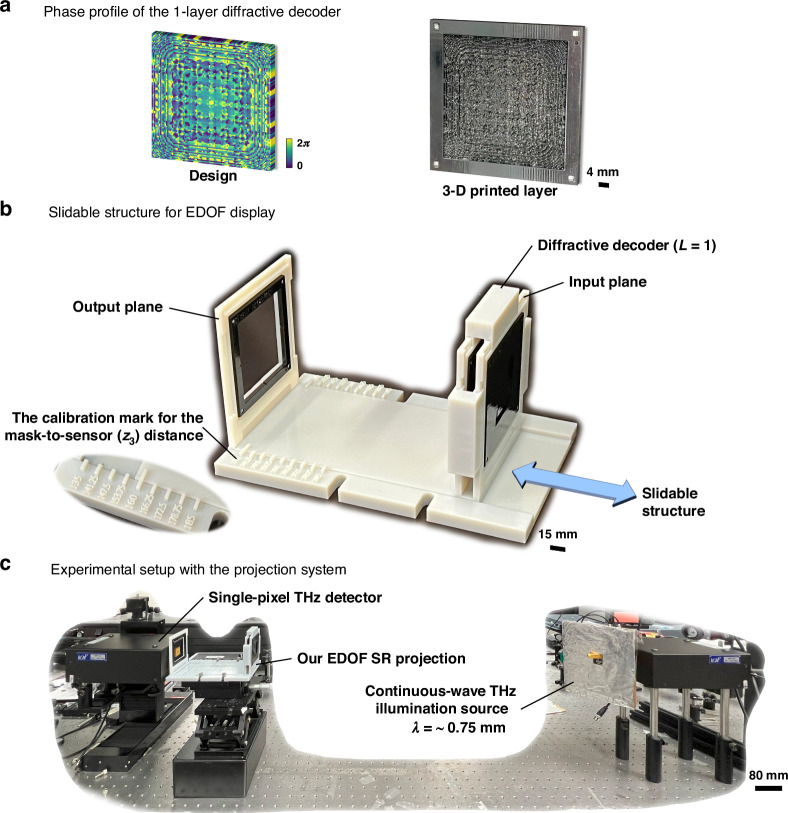
Experimental setup for EDOF PSR image projection system with *L=*1. **a** The phase profile of the designed diffractive decoder and its 3D fabricated version. **b** This 3D printed structure (white color) facilitates the relative axial translation of the diffractive decoder with respect to the terahertz detector at the output plane, enabling a variable layer-to-sensor distance to measure the resulting image projections at different axial positions. A calibration mark is integrated into the structure to ensure accurate measurement of the ${z}_{3}$ distance. **c** Experimental setup employing a continuous wave terahertz illumination and a THz detector

**Fig. 6:**
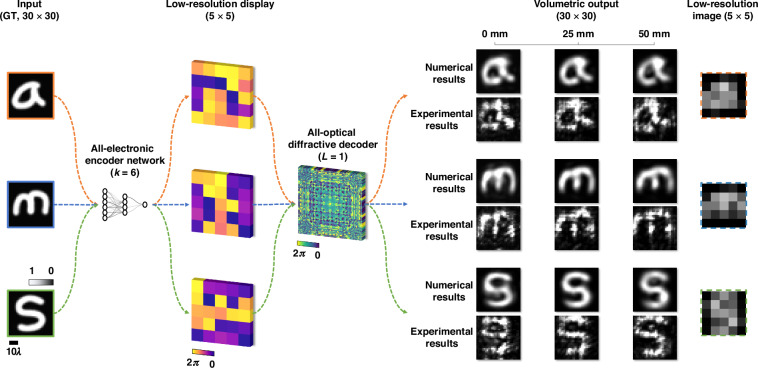
Experimental results of the extended DOF PSR image projection system in the terahertz spectrum. The hybrid system combines an all-electronic encoder network ($k=6$) with an all-optical diffractive decoder ($L=1$). Input images ($30\times 30$ pixels) are first encoded into low-resolution (5×5 pixels) phase patterns, which are then processed by the diffractive decoder through wave propagation. The EDOF output ($30\times 30$ pixels) shows both the numerical and the experimental images at selected depths of $0\,{\rm{mm}}$, $25\,{\rm{mm}}\,( \sim 33.36{\rm{\lambda }})$, and $50\,{\rm{mm}}\,( \sim 66.71{\rm{\lambda }})$, over a continuous projection. The rightmost column shows the corresponding low-resolution ($5\times 5$ pixels) images for comparison

In addition to these numerical analyses, our EDOF PSR image projection approach was also validated in proof-of-concept experiments spanning the terahertz and visible parts of the spectrum, demonstrating the scalability and generality of the hybrid system across different wavelengths. Specifically, for the experimental demonstration in the terahertz band (λ ≈ 0.75 mm), we designed a model with a single-layer diffractive decoder trained for ${z}_{3}^{{tr}}=135-185\,{\rm{mm}}\,(180.12{\rm{\lambda }}-246.84{\rm{\lambda }})$, corresponding to a ${z}_{e}=50\,\rm{mm}\,\left( \sim 66.71\lambda \right)$ and a PSR factor of $k=6$. A single diffractive layer was chosen to minimize performance degradation from potential inter-layer misalignments, which can be an important error source in experimental implementations, particularly for cascaded diffractive systems. To further enhance robustness and practical feasibility of our design, an additional power efficiency-related loss term was used, and random displacements (see the Methods section for details) were applied to the diffractive decoder surface during the training phase. As depicted in Fig. 5, we used a THz detector and a continuous-wave THz illumination source in our experiments after the 3D fabrication of the single-layer diffractive surface and the LR encoded-phase profiles, all of which were fabricated using a 3D printer (see the “Methods” section). Figure 6 illustrates the experimental and the corresponding numerical results for different positions ${(z}_{3}^{{te}})$ of the output plane, including $135\,{\rm{mm}}\,\left( \sim 180.12{\rm{\lambda }}\right)$, $160\,{\rm{mm}}\,\left( \sim 213.48{\rm{\lambda }}\right)$, and $185\,{\rm{mm}}\,( \sim 246.84{\rm{\lambda }})$. Collectively, these results confirm the system’s capability to deliver consistent, pixel super-resolved projections over an extended axial range in the terahertz domain.

**Fig. 7:**
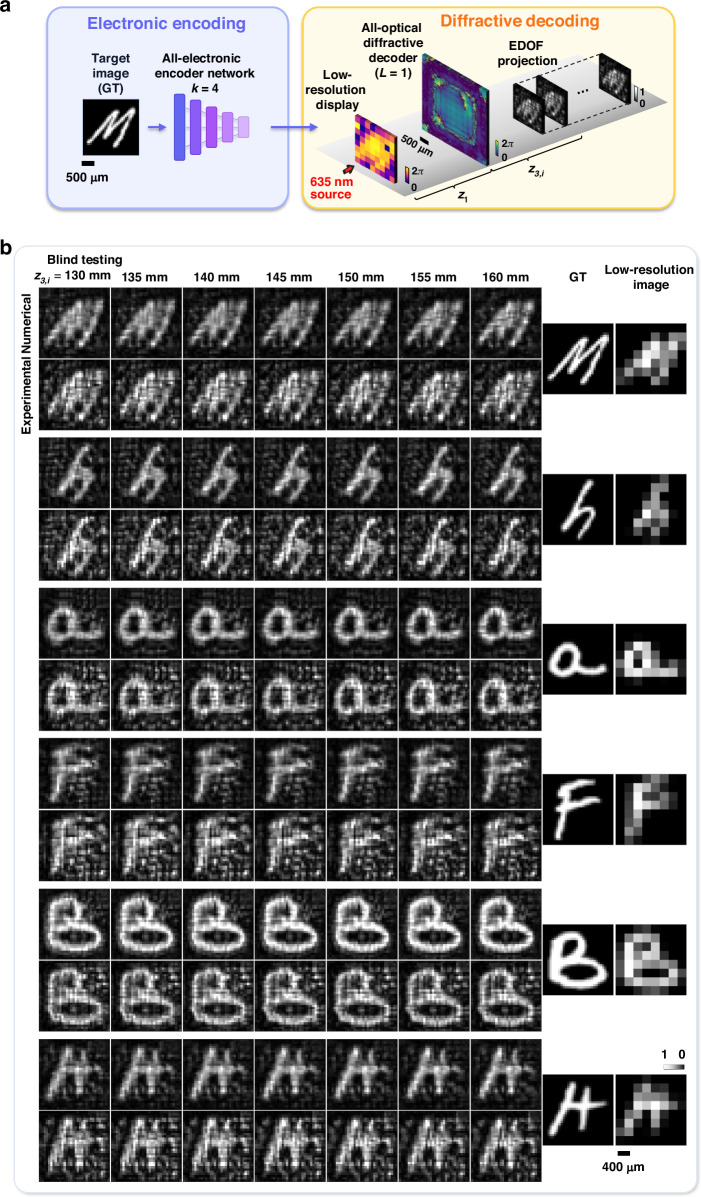
Experimental results of extended DOF PSR image projection in the visible spectrum. **a** Schematic of the hybrid system operating at 635 nm, comprising a CNN-based electronic encoder ($k=4$) and a single-layer ($L=1$) passive diffractive decoder. **b** Blind testing results for EMNIST characters never seen during the training. For each character, numerical (upper) and experimental (lower) output images are shown at seven axial planes spanning ${z}_{3,i}=130 - 160\ {\rm{mm}}$, demonstrating an extended DOF of ~30 mm. Ground truth (GT) images and the corresponding low-resolution inputs are displayed on the right side for comparison

To further validate the scalability and versatility of our hybrid framework, we conducted an additional proof-of-concept experiment in the visible range ($\lambda =635$ nm). As illustrated in Fig. 7a, this implementation employed a CNN-based electronic encoder with a super-resolution factor of $k=4$ and a single-layer passive diffractive decoder ($L=1$). In this visible-light setup, we employed two spatial light modulators (SLMs) to display the encoded phase patterns and the static diffractive decoder layer, respectively, axially separated by 17.5 mm (see the Methods section). While an SLM was used to implement the diffractive decoder in this proof-of-concept experiment to facilitate testing of different designs, it functioned strictly as a static phase mask, emulating a passive optical layer that can be fabricated using, e.g., lithography. Similar to the terahertz configuration, the system was jointly optimized to project super-resolved images across an axial range of ${z}_{3}\in \left[\mathrm{130,160}\right]\mathrm{mm}$. During the training phase, a random displacement strategy was applied to ensure robustness against potential misalignments (see the Methods section). Figure 7b presents the blind testing results for six randomly selected EMNIST characters, comparing numerical diffractive outputs with experimentally measured images across seven axial planes spanning the target volume. The experimental measurements exhibit good agreement with the simulations, maintaining high-fidelity image features over an extended DOF of 30 mm. These results, as shown in Figs. 6, 7, confirm the system’s capability to synthesize super-resolved projections over an extended DOF significantly larger than the illumination wavelength. Given the structural simplicity of the single-layer diffractive decoder, the results further highlight the framework’s efficiency in delivering substantial improvements in resolution and image projection depth with minimal optical hardware complexity.

**Fig. 8:**
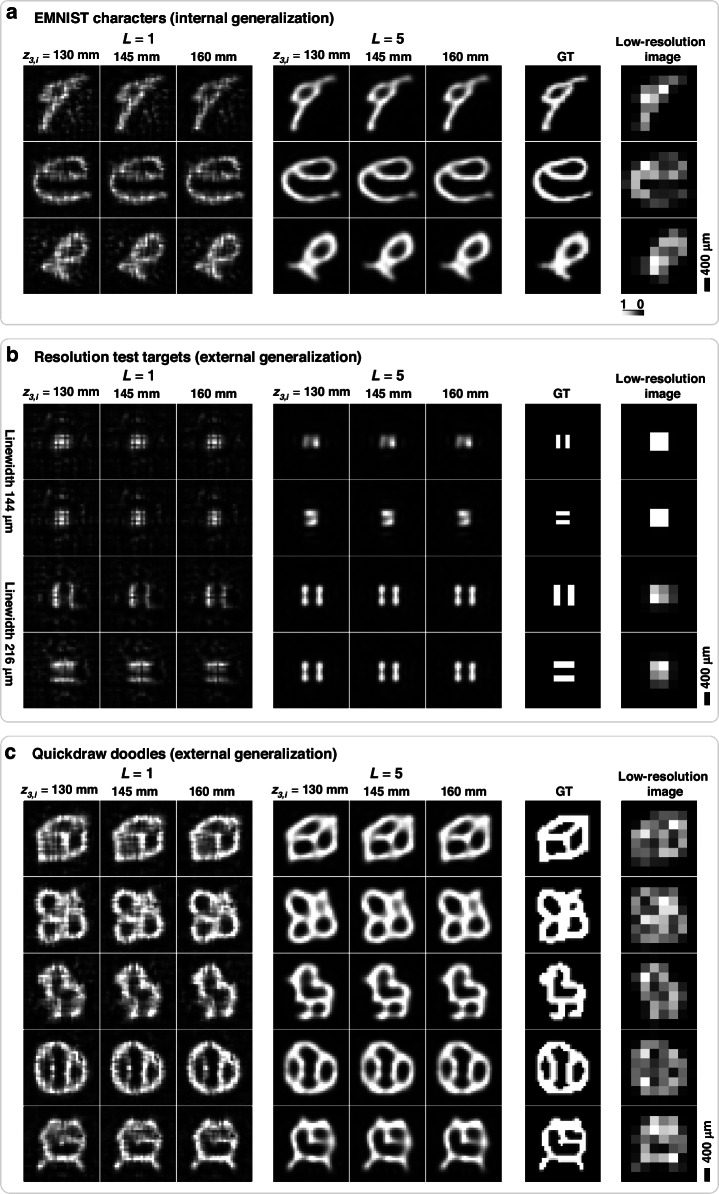
Generalization performance analysis of the extended DOF PSR image projection system in the visible spectrum. Numerical simulations of PSR ($k=4$) image projection operating at 635 nm, comparing single-layer ($L=1$) and multi-layer ($L=5$) diffractive decoders over an extended DOF (${z}_{3,i}=130 - 160\ {\rm{mm}}$. Both models were trained independently using exclusively the EMNIST dataset. **a** Internal generalization results on unseen EMNIST characters. **b** External generalization on resolution test targets. **c** External generalization on Quickdraw doodles. Ground truth (GT) images and the corresponding low-resolution inputs are displayed on the right side for comparison

Notably, our hybrid system is not tailored to a specific type of features or simple patterns to be projected, but functions as a general-purpose image projection platform. To demonstrate the versatility of our hybrid approach, extending beyond the testing on unseen EMNIST characters (Fig. 8a), we further numerically tested the external generalization capability of our hybrid system using image types/classes absent from training: resolution test targets (Fig. 8b) and Quickdraw doodles (Fig. 8c). For this study, we optimized a five-layer ($L=5$) diffractive design based on visible illumination ($\lambda =635\,{\rm{nm}}$) and compared it with the performance of a single-layer ($L=1$) diffractive design that was used in Fig. 7. Both models share an identical CNN encoder architecture and were trained end-to-end independently using exclusively the EMNIST dataset to achieve 4× PSR over an extended DOF of 130–160 mm. As shown in Fig. 8, both models demonstrate robust, consistent performance across diverse datasets. Quantitatively, the $L=5$ model achieved high fidelity on the internal generalization task (unseen EMNIST images), with an average test PSNR of 23.89±2.72 dB and an SSIM of 0.793±0.105. Regarding external generalization to new classes of test objects, evaluation on 5000 Quickdraw samples (50 randomly selected images from each of 100 test categories) yielded an average test PSNR of 19.13±1.69 dB and an SSIM of 0.724±0.070. We observed that the $L=5$ configuration achieves lower background noise and enhanced contrast compared to the $L=1$ model, indicating that additional passive diffractive layers effectively increase the accessible optical degrees of freedom and further improve image projection quality across various types of test objects. To quantitatively substantiate the super-resolution capability of our designs, further analyses, reported in Supplementary Figs. S7 and S8, show the spatial-frequency spectra and the cross-sections of the projected images. The radially averaged power spectra demonstrate that both the $L=1$ and $L=5$ decoders successfully recover high-frequency components extending beyond the Nyquist sampling limit of the low-resolution input display. While the single-layer ($L=1$) projections successfully retrieve the spatial-frequency spectra (Supplementary Fig. S7), their limited optical degrees of freedom result in residual noise and localized distortions. In contrast, the $L=5$ architecture (Supplementary Fig. S8) refines this spectral recovery, yielding cleaner 2D spatial spectra and sharper spatial cross-sections without the visual artifacts of the $L=1$ setup. Collectively, these results confirm that the encoder–decoder architecture learns a broadly applicable super-resolution mapping rather than memorizing dataset-specific features, thereby establishing our hybrid system as a generalized PSR image projection platform.

## Discussion

We presented a deep learning-enabled hybrid image projection system with enhanced DOF and resolution. This diffractive image projection system, integrating a CNN-based digital encoder and an all-optical passive decoder, reduces computational and hardware costs across various projection distances for a single wavefront modulator while also enhancing both the DOF and the spatial resolution. Moreover, this framework enhances data storage and transmission efficiency by encoding image information into compact phase patterns with significantly compressed dimensions. These patterns are subsequently processed by an all-optical decoder, resulting in the projection of high-resolution images with extended DOF. Specifically, the projected content can be dynamically updated via a compact display engine at the input encoded phase plane, such as an SLM, while the all-optical decoder part can be fabricated using passive optical materials. Notably, the overall image projection performance is not constrained by the nominal pixel count or the native SBP of the SLM itself. Instead, by introducing additional diffractive layers at the optical decoder, our hybrid system effectively surpasses the native SBP of the input display. This architecture enables the synthesis of high-fidelity projections with enhanced spatial resolution over an extended DOF.

One key strength of the proposed diffractive PSR framework lies in its inherent scalability, enabled by the passive nature of the optical decoder. The effective output resolution and the corresponding SBP can be systematically increased within the diffraction limit of light by scaling the number of diffractive features or layers, with or without increasing the number of pixels on the encoded pattern. As shown in the analysis in Fig. 8, increasing the number of diffractive features or layers consistently improves the output image fidelity. Accordingly, the number of diffractive layers or features can be tailored to the target image scales and/or performance requirements. Beyond a single device, the proposed framework naturally supports massive parallelization. Arrays of diffractive processors can be fabricated and operated concurrently, potentially enabling hundreds of passive optical decoders to function in parallel. Each diffractive unit can be designed to handle a distinct spatial sub-aperture, depth segment, or projection task, thereby further extending the effective SBP and throughput of the overall system.

Our hybrid approach also synergizes seamlessly with recent advances in deep learning–based CGH for large-scale display and image projection systems. CNN-based hologram generation methods have demonstrated the ability to map target images directly to phase-only holograms in a fast, non-iterative manner^12,14,32,33^, significantly reducing computational overhead compared to traditional CGH algorithms. Importantly, state-of-the-art CNN-based CGH models can be readily integrated into our architecture as digital encoders, enabling learned hologram synthesis to directly benefit from the physical SBP and DOF expansion provided by a jointly trained diffractive decoder. Due to computational resource limitations, the experimental results presented in this work use reduced-scale datasets and system sizes to demonstrate a proof of concept. Nevertheless, the underlying framework and training methodology are inherently scalable and can be designed for larger resolutions, deeper projection volumes, and higher SBP regimes.

Furthermore, precise alignment of the diffractive layers of the decoder can pose additional challenges for operation at shorter wavelengths. Some of these challenges can be mitigated by optimizing misalignment-resilient designs, implemented through a numerical “vaccination” design strategy^34,35^, by introducing potential errors and variations in, e.g., size/shape/orientation of the diffractive layers during the training process. The same approach can also be applied to reduce sensitivity to potential fabrication errors in the phase modulation features of the resulting diffractive layers. To further assess the feasibility of fabricating the designed multi-layer architectures, we investigated the resilience of our hybrid system against mechanical misalignments between the diffractive layers and the input plane; see Supplementary Figs. S4 and S5. Based on a three-layer decoder configuration ($L=3$) in Fig. 2, which was designed for a PSR factor of k = 6 trained for an extended DOF of ${z}_{e}=200\,{\rm{mm}}$, we evaluated the image projection fidelity under varying degrees of positional errors. Additionally, we employed a vaccination strategy, introducing random lateral ${(D}_{x} \sim U\left(-{\Delta }_{{xy},{tr}},{\Delta }_{{xy},{tr}}\right),$ ${D}_{y} \sim U\left(-{\Delta }_{{xy},{tr}},{\Delta }_{{xy},{tr}}\right))$ and axial (${D}_{z} \sim U\left(-{\Delta }_{{z},{tr}},{\Delta }_{{z},{tr}}\right)$) displacements to the diffractive layers during the training phase (see the Methods section for details). As shown in Supplementary Fig. S4a, the unvaccinated baseline diffractive model (${\Delta }_{{xy},{tr}}=0$) exhibits a decline in the output PSNR values from ~15.6 dB to ~11.5 dB as the testing misalignment increases, resulting in image degradation. Conversely, the models trained with vaccinated lateral shifts (${\Delta }_{{xy},{tr}}=0.2{\rm{\lambda }}$ and $0.4{\rm{\lambda }}$) demonstrate remarkable robustness, maintaining PSNR values above 15 dB even under significant lateral displacements up to $\sim \pm 0.5\lambda$. As visualized in Supplementary Fig. S4b, when ${\Delta }_{{xy},{test}}=0.6\lambda$, the output image PSNR value for the vaccinated design (${\Delta }_{{xy},{tr}}=0.4{\rm{\lambda }}$) remains at 15.29 dB, while the PSNR value for the baseline design (${\Delta }_{{xy},{tr}}=0$) drops to 12.32 dB. A similar trend is also observed for axial misalignments as shown in Supplementary Fig. S5, where the models vaccinated with ${\Delta }_{z,{tr}}=0.4{\rm{\lambda }}$ and $0.8{\rm{\lambda }}$ effectively mitigate the performance degradation associated with inter-layer spacing errors, reducing the output image PSNR degradation to ~0.1 dB. Crucially, this enhanced robustness incurs only a negligible penalty (~0.09 dB) on the peak image quality under ideal alignment conditions. These findings confirm that incorporating stochastic 3D positional noise during training effectively desensitizes the diffractive decoder design to uncontrolled mechanical variations, thereby relaxing stringent fabrication tolerances and facilitating robust experimental deployment of the EDOF PSR system.

In addition to alignment imperfections, fabrication constraints introduce another practical challenge, which is particularly critical for visible-wavelength applications employing lithography-based manufacturing. Specifically, current fabrication technologies typically limit the achievable axial thickness modulation of each diffractive layer, resulting in finite phase quantization per diffractive feature, which can lead to performance degradation relative to the ideal numerical designs. To systematically assess the robustness of our hybrid image projection framework under a limited phase bit-depth, we evaluated the system performance using phase bit depth values selected from $B\in \left\{\mathrm{16,8,6,5,4,3},\,2\right\}$. As shown in Supplementary Fig. S6a, when a diffractive model is trained using a large phase bit-depth (e.g., ${B}_{{train}}=16$), the image projection performance gradually degrades as the phase quantization level is reduced during the testing (${B}_{{test}}\in\left\{\mathrm{16,8,6,5,4,3,2}\right\}$). For instance, the average output image PSNR decreases from ~15.8 dB at ${B}_{{test}}=16$ to ~ 14.4 dB at ${B}_{{test}}=3$, reflecting the increasing phase discretization error. Importantly, this degradation can be effectively mitigated by incorporating the phase quantization constraint directly into the training process. When the diffractive processor is trained and optimized with a reduced phase resolution (e.g., ${B}_{{train}}=3$) and evaluated using the same bit depth (${B}_{{test}}={B}_{{train}}=3$), the average output PSNR improves to ~15.6 dB, approaching the performance of higher phase bit-depth designs. The corresponding visual reconstructions shown in Supplementary Fig. S6b further demonstrate consistent image quality, preserved contrast, and stable feature resolution across the entire image-projection depth range, despite severe phase quantization. Together, these results highlight the robustness of our hybrid diffractive framework, confirming that high-fidelity and large-DOF image projection can be achieved even under stringent fabrication constraints by co-designing the training process with realistic hardware limitations in mind.

Further advances in this technology promise to unlock new paradigms across diverse fields ranging from display technologies to advanced scientific instrumentation and optical metrology. For example, deep learning-based metrology methods have recently attracted considerable attention^36^, owing to their advantages over traditional model-based approaches. Our diffractive image projection system, combining a CNN-based encoder with an all-optical decoder, might potentially offer new types of solutions to optical metrology tasks.

Notably, the presented extended DOF PSR image projection approach can operate across different parts of the electromagnetic spectrum. The designed models can be scaled to any part of the spectrum, including the visible and infrared (IR) wavelengths, by physically scaling the dimensions of the spatial features with respect to the wavelength of light. Crucially, recent advances in nano-fabrication techniques provide a practical pathway toward realizing large-scale diffractive decoders for visible display applications. State-of-the-art fabrication techniques, including advanced optical lithography and electron-beam lithography^37–40^, enable lateral feature sizes on the order of a few hundred nanometers, axial step sizes below 100 nanometers, and precise multi-level phase modulation, all readily applicable to visible holographic display applications. Furthermore, wafer-level processes have been demonstrated to support hundreds of millions to billions of nanoscale diffractive features per wafer^40^, with multi-layer alignment accuracy sufficient for complex optical processing tasks. These capabilities enable the fabrication of large-aperture, multi-layer diffractive decoders with high uniformity, repeatability, and compatibility with mass production. Once integrated with these manufacturing capabilities, our hybrid electronic-optical approach can establish a compelling route toward high-resolution image projection and display platforms that surpass the SBP and power-efficiency limits of conventional holographic displays.

In summary, our EDOF PSR image projection system, enabled by the symbiosis of deep learning with diffractive optical processors, represents a significant advance toward versatile and high-performance light shaping, with far-reaching implications for fields that demand precise lateral and depth control of light, including microscopy and metrology.

## Materials and methods

### Digital encoder design

Our digital encoder, depicted in Fig. 1a, consists of a series of convolutional layers followed by a fully connected layer. It accepts a $24\times 24$ grayscale image as input. Each convolutional layer applies its filters to the input, followed by normalization and a PReLU^25^ activation. Max-pooling operations between the layers progressively reduce the spatial dimensions while increasing the feature depth. As the input progresses through the network, the spatial dimensions are reduced while the number of channels increases. The final convolutional layer yields a $3\times 3\times 128$ feature map, which is then flattened and processed by a fully connected layer with sigmoid activation, producing a compact encoded representation ($\frac{24}{k}\times \frac{24}{k}$ pixels, where a PSR factor of $k=6$ is used in Fig. 1a). This encoding operation enables the digital encoder to efficiently compress the essential information of the input image into a phase-only format optimized for subsequent optical decoding.

### Optical decoder design

Our diffractive decoder employs passive diffractive layers, $L\ge 1$, and each layer $l$ is then subdivided into a grid of diffractive features^22^^,41^^,42^^,43^. These diffractive features, characterized by their transmittance coefficients ${t}_{l}[m,n]$, serve as the fundamental building blocks of the all-optical decoder. The transmittance of an individual diffractive feature is defined as follows:

1 $${t}_{l}\left[m,n\right]=\exp \left(j\frac{2{\rm{\pi }}}{{\rm{\lambda }}}\left({\rm{\tau }}\left({\rm{\lambda }}\right)-{n}_{a}\right){h}_{l}\left[m,n\right]\right)$$

In this expression, the wavelength-dependent complex refractive index $\tau (\lambda )=n\left(\lambda \right)+j\kappa \left(\lambda \right)$ and the refractive index of the surrounding medium ${n}_{a}$ determine the phase modulation imparted by each diffractive feature. A key parameter in the modulation process is the physical thickness of each diffractive feature, i.e., ${h}_{l}[m,n]$, which is computed using an auxiliary variable ${o}_{l}\left[m,n\right]$:

2 $${h}_{l}\left[m,n\right]=\frac{\tanh \left({o}_{l}\left[m,n\right]\right)+1}{2}\left({h}_{m}-{h}_{b}\right)+{h}_{b}$$

This formulation allows for a continuous spectrum of thickness values, bounded by ${h}_{b}$ and ${h}_{m}$, which can be tuned during the optimization process to achieve desired optical properties. These diffractive features give rise to a transmission modulation function ${T}_{l}\left(x,y\right)$, described by:

3 $${T}_{l}\left(x,y\right)=\mathop{\sum }\limits_{m}\mathop{\sum }\limits_{n}{t}_{l}\left[m,n\right]{p}_{l}\left(x-m{W}_{x},y-n{W}_{y}\right)$$

where the 2D rectangular sampling kernel ${p}_{l}\left(x,y\right)$ is defined as:

4 $${p}_{l}\left(x,y\right)=\left\{\begin{array}{l}1,\,\left|x\right| < \frac{{W}_{x}}{2},\left|y\right| < \frac{{W}_{y}}{2}\\ 0,\,\rm{otherwise}\end{array}\right.$$

To model the propagation of spatially and temporally coherent light propagating through this system, the framework employs the angular spectrum method^42^, which is based on the fast Fourier transform-based implementation of the Rayleigh-Sommerfeld diffraction integral. This propagation is mathematically expressed as:

5 $${U}_{l+1}\left(x,y\right)={{U}^{{\prime} }}_{l}\left(x,y\right)* w\left(x,y,{z}_{l+1}-{z}_{l}\right)$$

where ${{U}^{{\prime} }}_{l}\left(x,y\right)={U}_{l}\left(x,y\right){T}_{l}\left(x,y\right)$ is the output field of layer $l$, and the term ${z}_{l+1}-{z}_{l}$ represents the axial propagation distance between the diffractive layer $l$ and the next diffractive layer $l+1$. The propagation kernel $w\left(x,y,z\right)$ is given by:

6 $$w\left(x,y,z\right)=\frac{z}{{r}^{2}}\left(\frac{1}{2{\rm{\pi }}r}+\frac{1}{j{\rm{\lambda }}}\right)\exp \left(j\frac{2{\rm{\pi }}r}{{\rm{\lambda }}}\right)$$

with $$r=\sqrt{{x}^{2}+{y}^{2}+{z}^{2}}$$.

### Datasets and image preprocessing

The EMNIST handwritten letters dataset, consisting of 88,800 training and 14,800 testing samples, was used to train our numerical designs. We allocated 8800 samples from the training set for validation, yielding 80,000 training, 8800 validation, and 14,800 testing samples. Handwritten letter images ($28\times 28$ pixels) from this dataset were resized to $24\times 24$ pixels for terahertz experiments and $32\times 32$ pixels for visible-light experiments using bicubic downsampling. For the experimental phase, we utilized the same dataset to train the hybrid image projection system. To evaluate its performance, we tested the model using handwritten letter images that are different from those in the training dataset.

For external generalization testing, we utilized the ‘Quick, Draw!’ dataset^44^, selecting 5000 images (50 randomly chosen samples from 100 different categories). These images were resized to $32\times 32$ pixels and subjected to a max-pooling operation with a $3\times 3$ kernel during preprocessing. This dataset was used solely for testing and was not included in any part of the model’s training process.

### Optical decoder parameters

Our numerical designs in the terahertz spectrum incorporated diffractive layers spanning $133.42\lambda \times 133.42\lambda$, each discretized into a $200\times 200$ element grid. The size of diffractive features and the sampling period of the propagation model were set to $0.667\lambda$. The input and output fields of view of the decoder are $96\times 96$ pixels ($64.04\lambda \times 64.04\lambda$), and aliasing was prevented through zero padding to $400\times 400$ pixels. The transmittance coefficient of each diffractive element was expressed as:

7 $${t}_{l}\left[m,n\right]=\exp \left(j{{\rm{\theta }}}_{l}\left[m,n\right]\right)=\exp \left(j\frac{2{\rm{\pi }}}{{\rm{\lambda }}}\left(n\left({\rm{\lambda }}\right)-{n}_{a}\right){h}_{l}\left[m,n\right]\right)$$

Here, the phase coefficients ${\theta }_{l}\left[m,n\right]$ were initialized to zero and subsequently refined through stochastic gradient-based error backpropagation^45^.

We explored different decoder configurations with *L*=1, 3 and 5 transmissive layers; for comparison, we also trained $L=0$ designs without a diffractive decoder layer. By utilizing a grid search method, we determined the optimal distance from the input plane to the first diffractive layer (${z}_{1}$), the inter-layer spacing between the diffractive layers (${z}_{2}$), and the distance from the final layer to the first output plane (${z}_{\mathrm{3,1}}$), as illustrated in Fig. 1b and Table 1. The final configuration was determined by maximizing the average peak signal-to-noise ratio (PSNR) across the validation dataset, ensuring optimized and robust performance under varied input conditions. By empirically optimizing both the axial placement and the number of diffractive layers, the presented hybrid diffractive system effectively exploits the available optical degrees of freedom, leading to improved image projection performance.

**Table 1:** The axial distances used for the numerical and experimental evaluations of the diffractive decoders reported in this work

Distance	${z}_{1}$	${z}_{2}$	${z}_{\mathrm{3,1}}$	${z}_{e}$
Numerical	$10\,\rm{mm}\,\left(13\lambda \right)$	$50\,\rm{mm}\,\left(66\lambda \right)\,$	$150\,\rm{mm}\,\left(200\lambda \right)$	$200\,\rm{mm}\,\left(267\lambda \right)$
Experimental	$10\,\rm{mm}\,\left(13\lambda \right)$	N/A	$155\,\rm{mm}\,\left(207\lambda \right)$	$50\,\rm{mm}\,\left(66\lambda \right)$

In our experimental results in the terahertz spectrum, a monochromatic terahertz source emitting at $\lambda \approx 0.75\,\rm{mm}$ was used. Our experiments focused on a single-layer ($L=1$) diffractive decoder, with specific axial distances also detailed in Table 1.

The diffractive layers were constructed using diffractive features, each with dimensions of $0.667{\rm{\lambda }}$, arranged in a $66.7{\rm{\lambda }}\times 66.7{\rm{\lambda }}$ grid ($50\,{\rm{mm}}\times 50\,{\rm{mm}}$). Similarly, we established an effective pixel size of $1.33{\rm{\lambda }}$ at the measurement plane, while both the phase-only wavefront modulator and the output field of view were designed as $40{\rm{\lambda }}\times 40{\rm{\lambda }}$ ($30\ {\rm{mm}}\times 30\ {\rm{mm}}$). The LR wavefront modulator’s pixel pitch was set to $8{\rm{\lambda }}\times 8{\rm{\lambda }}$, yielding a PSR factor of 6.

The diffractive components were fabricated using a 3D-printing material characterized by a complex refractive index of ${\rm{\tau }}\left({\rm{\lambda }}\right)\approx 1.6518+j0.0612$ at the operational wavelength, $\lambda \approx 0.75\,\rm{mm}$. The thickness of each diffractive feature, denoted as ${h}_{l}\left[m,n\right]$, was optimized within the range of $\left[0.5\,{\rm{mm}},1.644\,{\rm{mm}}\right]$, corresponding to a phase modulation span of $\left[-{\rm{\pi }},{\rm{\pi }}\right)$.

For the visible PSR EDOF projection design reported in Fig. 7a, we utilized phase-only SLMs and treated the diffractive component, including the encoded phase profile and the diffractive layer, as phase modulators, with a reflection coefficient of:

8 $$r\left({x}_{s},{y}_{s},{z}_{l};\lambda \right)=\exp \left(-j\phi ({x}_{s},{y}_{s};\lambda )\right)$$

where *ϕ*(*x*_*s*_,*y*_*s*_;*λ*) is the phase modulation value in the range of $\left[-{\rm{\pi }},{\rm{\pi }}\right)$ for the corresponding diffractive feature. The diffractive features have dimensions of 4.5 µm, arranged in an $800\times 800$ pixel grid. Both the phase-only wavefront modulator and the output field of view were designed as $3628\lambda \times 3628\lambda$ ($2.304\times 2.304\,\rm{mm}$). The LR wavefront modulator’s pixel pitch was set to $441\lambda \times 441\lambda$ ($0.288\times 0.288\,\rm{mm}$), yielding a PSR factor of k = 4.

To enhance the robustness of our system, we implemented a vaccination process^34^ during the training. This involved introducing random alignment errors to the positions of diffractive layers and the input patterns during the training phase. The random errors are represented by ${D}^{l}=\,\left({D}_{x},\,{D}_{y},\,{D}_{z}\right)$, indicating deviations of the diffractive layer $l$ from its ideal position. Here, ${D}_{x}$, ${D}_{y}$, and ${D}_{z}$ independently follow a uniform distribution, such that ${D}_{x} \sim U\left(-{\Delta }_{x},{\Delta }_{x}\right)$, ${D}_{y} \sim U\left(-{\Delta }_{y},{\Delta }_{y}\right)$, and ${D}_{z} \sim U\left(-{\Delta }_{z},{\Delta }_{z}\right)$, where the parameters ${\Delta }_{x}$, ${\Delta }_{y}$, and ${\Delta }_{z}$ denote the hyperparameters of the possible alignment errors along the x-, y-, and z-axes, respectively. Consequently, the position of the diffractive layer $l$ at the ${i}^{\rm{th}}$ iteration, denoted as ${L}^{\left(l,i\right)}$, is expressed as:

9 $${L}^{\left(l,i\right)}=\left({L}_{x}^{l},{L}_{y}^{l},{L}_{z}^{l}\right)+\left({D}_{x}^{\left(l,i\right)},{D}_{y}^{\left(l,i\right)},{D}_{z}^{\left(l,i\right)}\right)$$

The alignment error ranges for our terahertz experiments were set to $0.67{\rm{\lambda }}$ for both $\Delta x$ and $\Delta y$, and $0.534{\rm{\lambda }}$ for $\Delta z$. For experiments in the visible spectrum, we set $\Delta z=0$ and $\Delta {x}=\,\Delta {y}=\,0.75{\rm{\lambda }}$.

Following the decoder optimization, we converted the thickness maps of the diffractive layers and the corresponding phase-encoded representations into STL files using MATLAB. These designs were then physically fabricated using an Objet30 Pro 3D printer (Stratasys). To precisely adjust the distance between the decoder and the fixed terahertz wave detector at the output plane, a slidable structure, illustrated in Fig. 5b. was designed and 3D-printed. This configuration enables output image measurements over an extended DOF by controlling the distance from the diffractive layer to the output plane (${z}_{3,i}$).

### Experimental setup

The experimental apparatus is depicted in Fig. 5c. At its core, a Virginia Diodes Inc. WR2.2 modular amplifier/multiplier chain (AMC) serves as the radiation source. This device transforms an 11.111 GHz (f_RF1_) input, amplified to 10 dBm, into a 0.4 THz continuous wave through a 36-fold frequency multiplication process. To enhance detection sensitivity, the output undergoes 1-kHz amplitude modulation. Terahertz waves emanate from a diagonal horn antenna, traversing 60 cm before encountering the target object and, subsequently, a 3D-printed diffractive decoder. A Virginia Diodes Inc. single-pixel Mixer/AMC, operating with an 11.083 GHz (f_RF2_) local oscillator at 10 dBm, captures and down-converts the diffracted field to a 1 GHz intermediate frequency. A detector ($0.5\,{\rm{mm}}\times 0.25\,{\rm{mm}}$) is mounted on Thorlabs NRT100 motorized linear stages, enabling precise X-Y positioning. It scanned the field of view in 0.75 mm steps, and subsequently, $3\times 3$ bilinear upsampling and 4 × 4-pixel binning were employed to increase the signal-to-noise ratio and approximate the system’s ~1.33λ (1 mm) output pixel size. Mini-Circuits ZRL-1150-LN+ amplifiers provide 40 dB gain, followed by a KL Electronics 3C40-1000/T10-O/O bandpass filter (1 GHz ± 10 MHz). An HP 8495B attenuator allows for system calibration before the signal reaches a Mini-Circuits ZX47-60 power detector. Final processing occurs in a Stanford Research SR830 lock-in amplifier synchronized to the 1 kHz modulation. The resulting data undergoes linear scaling and dynamic range adjustment, where the extreme 5% of values at both ends are saturated, with the remainder mapped to a 0-1 range.

For the PSR EDOF projection experiment shown in Fig. 7a, a laser source (Fianium Ltd, Southampton, UK) provided the illumination at 635 nm. Phase patterns were displayed onto two SLMs that served as the encoded phase pattern and the pre-trained diffractive layer: a HOLOEYE PLUTO-2.1 device (8 μm pixel pitch, 1920 × 1080) and a HOLOEYE LUNA device (4.5 μm pixel pitch, 1920 × 1080), respectively. A complementary metal-oxide-semiconductor (CMOS) camera (Basler ace acA1920-40gm) was used for capturing the output images. Light propagated $17.5\,{\rm{mm}}$ between the two SLMs and covered an output DOF of $\sim 30\,\rm{mm}$.

### Loss function and DOF-aware optimization strategy

The optimization of our EDOF PSR image projection system involves the joint training of an electronic CNN-based encoder and an optical diffractive decoder. We formulated a comprehensive loss function that balances the image projection accuracy with the output diffraction efficiency:

10 $${{\mathcal{L}}}_{{sample}}=\frac{1}{N}\mathop{\sum }\limits_{j=1}^{N}{\left({y}_{j}-{\rm{\sigma }}{\hat{y}}_{j,{z}_{3,i}}\right)}^{2}+{\rm{\alpha }}{e}^{-\eta }$$

This loss function comprises two key components: the mean squared error (MSE) between the target high-resolution image (${y}_{j}$) and the normalized decoder output at a randomly chosen ${z}_{3,i}$ distance ($\sigma {\hat{y}}_{j,{z}_{3,i}}$), and a diffraction efficiency-related penalty term (${e}^{-\eta }$). Here, *N* denotes the number of pixels in each image, $\sigma =\frac{{\sum }_{{{j}}=1}^{{{N}}}{y}_{j}\,}{{\sum }_{{{j}}=1}^{{{N}}}{\hat{y}}_{j,{z}_{3,i}}}$ represents a normalization factor, and α is a weighting coefficient for the diffraction efficiency-related term. During the numerical validations, we set $\alpha$ to 0, and in our experimental designs, we set $\alpha$ to 0.01 to achieve ~2% output diffraction efficiency. The diffraction efficiency term, η, is defined as $100\times \frac{{P}_{{out}}}{{P}_{{in}}}$, where ${P}_{{in}}$ and ${P}_{{out}}$ represent the input and output optical powers, respectively, and it enables fine-tuning of the diffractive decoder’s power efficiency through Eq. (10). For each training iteration, the total loss ${L}_{{batch}}$ is calculated as the mean of all ${L}_{{sample}}$ values within that batch.

To assess the quality of our projected output images, we employed two widely recognized metrics: SSIM^46^ and PSNR. SSIM evaluates the similarity between the reconstructed and the ground truth images using:

11 $${SSIM}\left(x,y\right)=\frac{\left(2{{\rm{\mu }}}_{x}{{\rm{\mu }}}_{y}+{c}_{1}\right)\left(2{{\rm{\sigma }}}_{{xy}}+{c}_{2}\right)}{\left({{\rm{\mu }}}_{x}^{2}+{{\rm{\mu }}}_{y}^{2}+{c}_{1}\right)\left({{\rm{\sigma }}}_{x}^{2}+{{\rm{\sigma }}}_{y}^{2}+{c}_{2}\right)}$$

where ${\mu }_{x}$ and ${\mu }_{y}$ are the mean values of image $x$ and $y$, ${\sigma }_{x}^{2}$ and ${\sigma }_{y}^{2}$ are the variances of image $x$ and $y$, ${\sigma }_{{xy}}$ is the covariance of $x$ and $y$, respectively, and ${c}_{1}$ and ${c}_{2}$ are constants for numerical stability. PSNR quantifies the ratio of the maximum signal power to noise power:

12 $${PSNR}=10{\log }_{10}\left(\frac{{MA}{X}_{I}^{2}}{{MSE}}\right)$$

with ${MA}{X}_{I}$ denoting the maximum possible pixel intensity.

Additionally, to evaluate the modulation depth of the projected patterns, we utilized the Michelson Contrast metric. Prior to the metric calculation, the projected image intensity $I$ is normalized using min-max normalization. The contrast is then computed based on the regional statistics defined by a binary target (ground truth) mask $T$:

13 $${MC}=\frac{{{\rm{\mu }}}_{w}-{{\rm{\mu }}}_{b}}{{{\rm{\mu }}}_{w}+{{\rm{\mu }}}_{b}+{\rm{\epsilon }}}$$

where ${{\rm{\mu }}}_{w}$ and ${{\rm{\mu }}}_{b}$ represent the mean pixel intensities of the bright regions (where the target mask $T=1$) and the dark regions (where $T=0$), respectively, and ${\rm{\epsilon }}$ is a small constant introduced to ensure numerical stability.

We implemented our training framework using Python 3.9.13 and TensorFlow 2.10, leveraging the Adam optimizer with a Cosine Annealing learning schedule incorporating a warm-up phase. The optimization process utilized a maximum learning rate of $1\times {10}^{-3}$, with 25 warm-up epochs and a total of 400 training epochs. All networks were trained on an NVIDIA GeForce RTX 3090 GPU, with each design requiring approximately 28 hours of training time using a batch size of 200.

## Supplementary information

Supplementary Information

## Data Availability

All the data and methods needed to evaluate the conclusions of this work are presented in the main text. Additional data can be requested from the corresponding author (A.O.). The codes used in this work use standard libraries and scripts that are publicly available in TensorFlow.
