# Supplementary material for: Super-resolution image projection over an extended depth of field using a diffractive decoder
Source: Light Sci Appl. 2026 May 18;15:236. doi: 10.1038/s41377-026-02320-7 (PMC13184326; doi:10.1038/s41377-026-02320-7)
Supplement: Supplementary file 1 — Supplementary Information [file 41377_2026_2320_MOESM1_ESM.pdf]

Supplementary Information for

Super-resolution image projection over an extended depth of field using a diffractive decoder

Hanlong Chen[1,2,3†], Çağatay Işıl[1,2,3†], Che-Yung Shen[1,2,3†], Shiqi Chen[1,2,3], Tianyi Gan[1], Mona Jarrahi[1], Aydogan Ozcan[1,2,3*]

1 Electrical and Computer Engineering Department, University of California, Los Angeles, California 90095, USA

2 Bioengineering Department, University of California, Los Angeles, California 90095, USA

3 California NanoSystems Institute (CNSI), University of California, Los Angeles, California 90095, USA

[†]These authors contributed equally to this work.

[*]Corresponding author: ozcan@ucla.edu

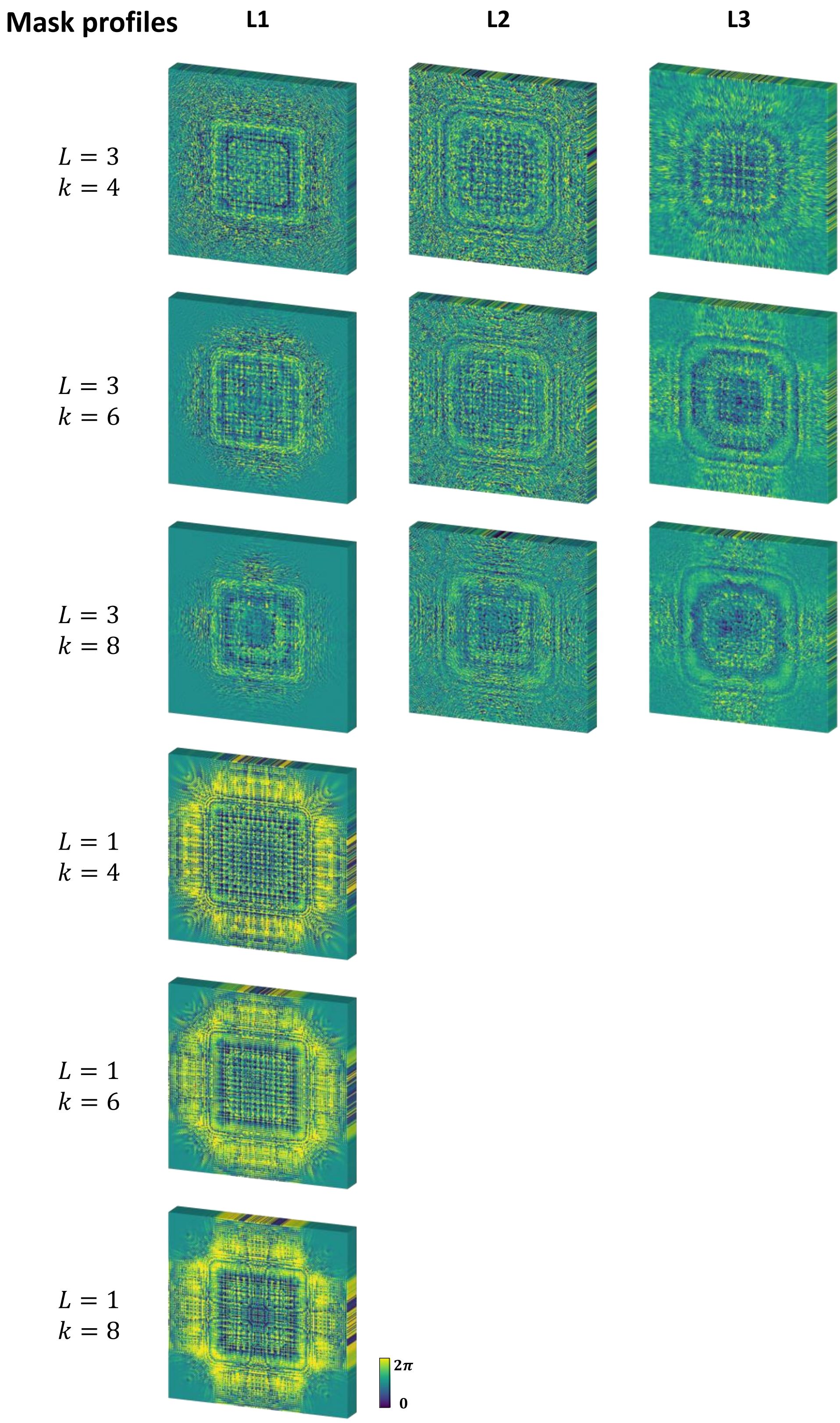

Supplementary Figure S1: Phase profiles of the diffractive layers employed by the all-optical diffractive decoders shown in Figures 1-4 of the main text.

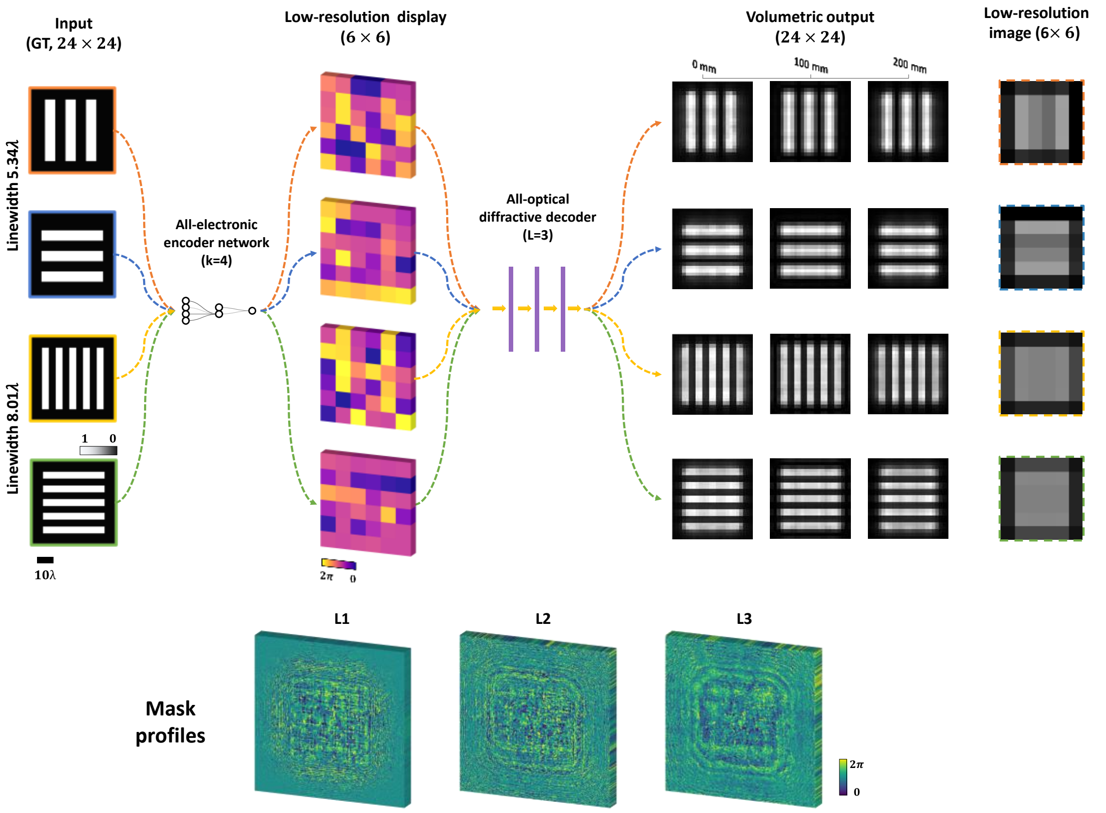

Supplementary Figure S2: Fringe projection results of the hybrid PSR image projection system. The input fringe images (24 × 24 pixels) with different linewidths (5.34λ and 8.01λ) are processed through a digital encoder network ($k = 4$) and a passive diffractive decoder with three layers ($L = 3$). Each row represents a distinct fringe pattern processed by the encoder-decoder pair. The low-resolution display images (6 × 6 pixels) are shown in the middle column, illustrating the encoded phase representations. The volumetric image outputs represent continuous projections with an EDOF of 200 mm ($\sim 266.85\lambda$), and cross-sectional views are displayed at various axial propagation distances, $z = 0$ mm (0λ), 100 mm ($\sim 133.43\lambda$), and 200 mm ($\sim 266.85\lambda$). The rightmost column presents the low-resolution images (6 × 6 pixels) corresponding to each input fringe pattern. The bottom row depicts the phase profiles of the diffractive decoder layers used in the decoder ($L1$, $L2$ and $L3$).

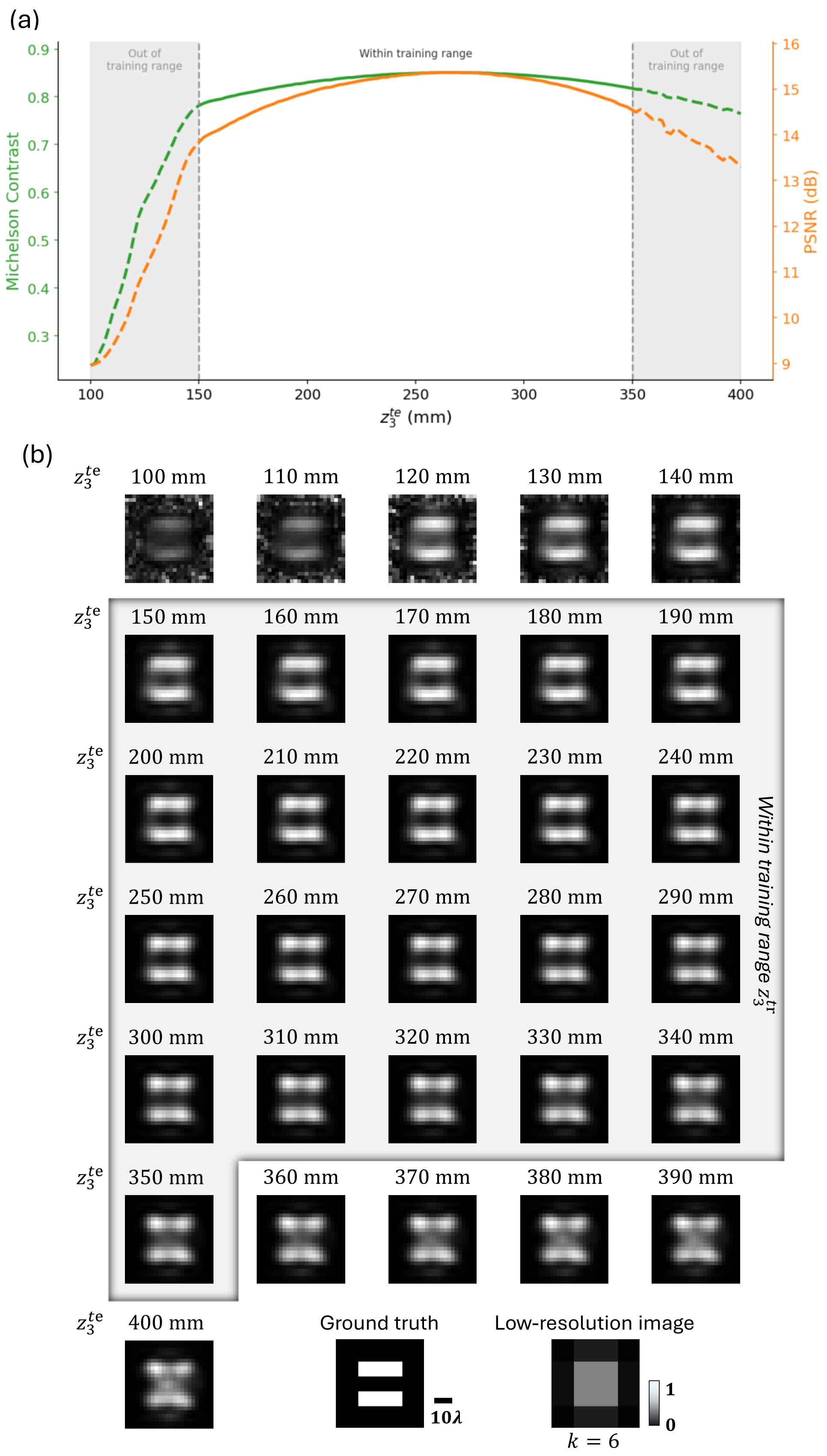

Supplementary Figure S3: Quantitative evaluation of the EDOF range of the hybrid PSR image projection system ($k = 6$) using resolution test targets. Grating

patterns (linewidth = 10.6λ), absent from the EMNIST training set, are used to quantify the system's EDOF performance. (a) Michelson contrast and PSNR values as a function of the testing projection distance z_3^{te}, evaluated using both horizontal and vertical gratings. The white region denotes the training range ($z_3^{tr} \in [150,350]$ mm), within which a stable high-contrast projection is achieved over an extended DOF. Shaded regions indicate out-of-training range. (b) Projected outputs of horizontal gratings sampled at z_3^{te} from 100 mm to 400 mm. Ground truth and corresponding low-resolution input images ($k = 6$) are also shown at the bottom for reference.

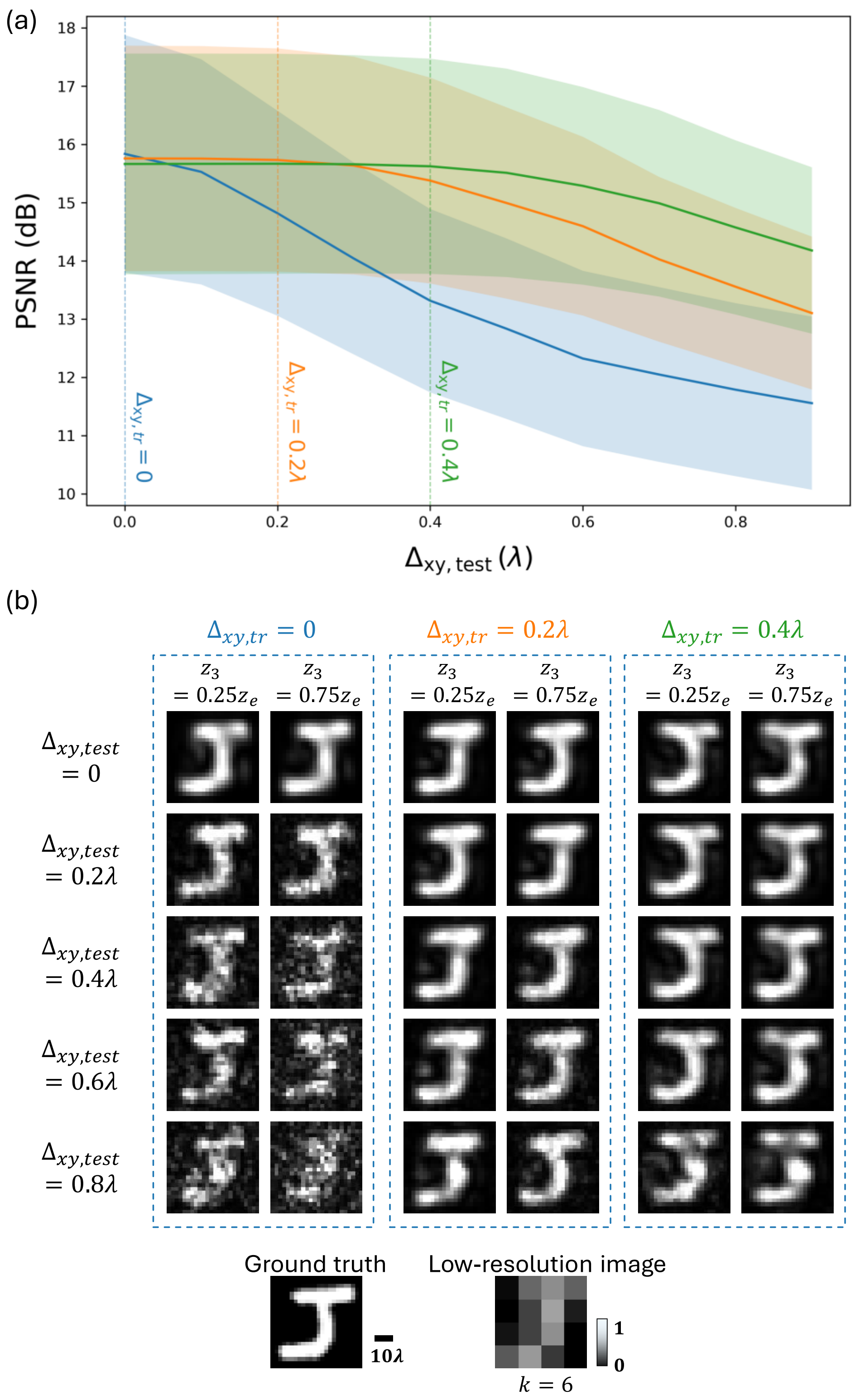

Supplementary Figure S4: Impact of lateral misalignments on the EDOF projection performance of the hybrid image projection system using $k = 6$, $L = 3$, $z_e = 200$ mm and $\lambda = 0.75$ mm. (a) Average PSNR (solid lines) and standard deviation values (shaded regions) are plotted as a function of the lateral positioning error ($\Delta_{xy,test}$). The colors correspond to models trained with different lateral vaccination levels ($\Delta_{xy,tr}$). (b) Projected images at two axial depths ($0.25z_e$ and $0.75z_e$)

under varying testing lateral misalignment conditions. The ground truth and the low-resolution input images are shown at the bottom for reference.

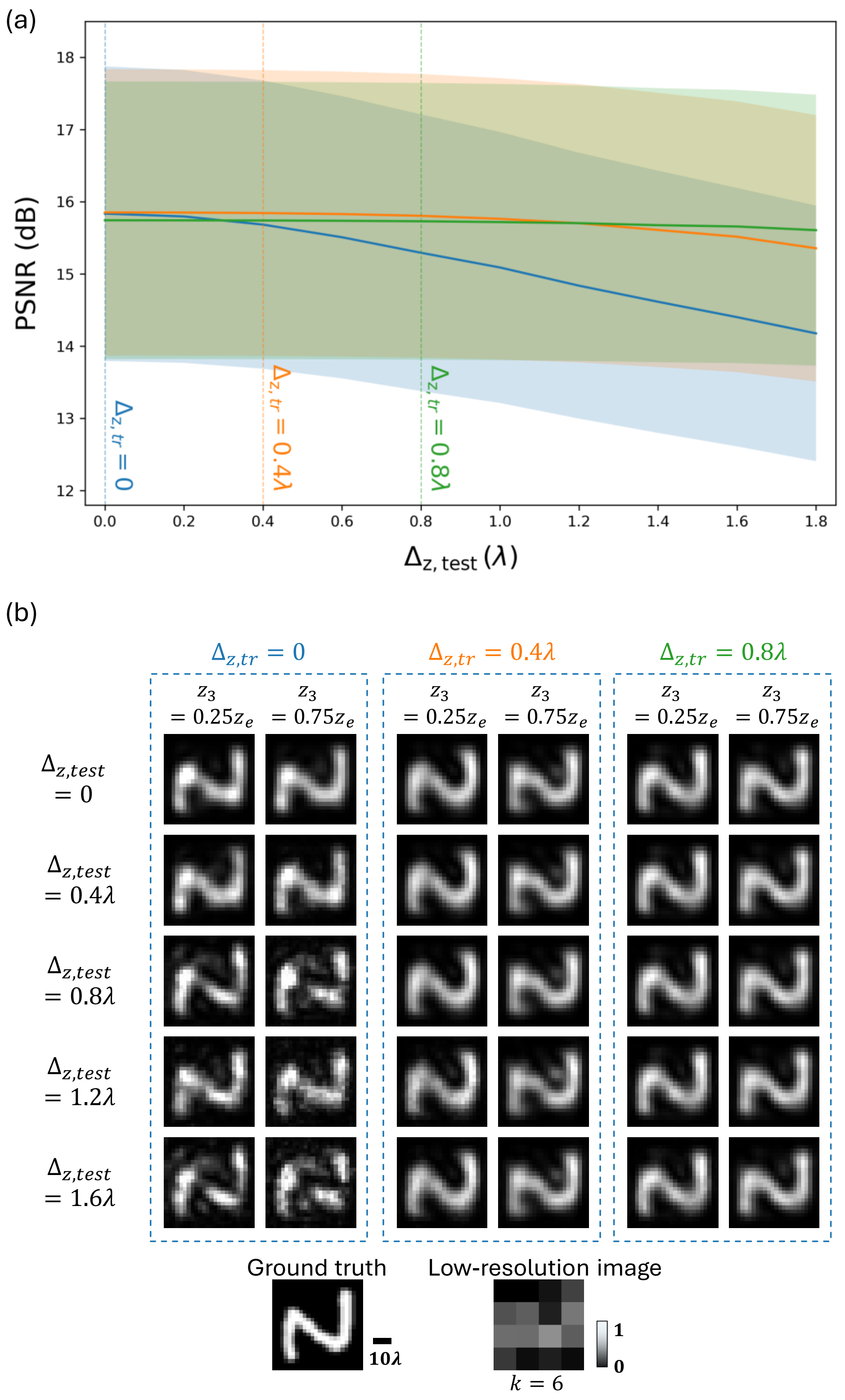

Supplementary Figure S5: Impact of axial misalignments on the EDOF projection performance of the hybrid image projection system using $k = 6$, $L = 3$, $z_e = 200$ mm and $\lambda = 0.75$ mm. (a) Average PSNR (solid lines) and standard deviation values (shaded regions) are plotted as a function of the axial positioning error ($\Delta_{z,test}$). The colors correspond to models trained with different axial vaccination levels ($\Delta_{z,tr}$). (b) Projected images at two axial depths ($0.25z_e$ and $0.75z_e$) under varying testing

axial misalignments. The ground truth and the low-resolution input images are shown at the bottom for reference.

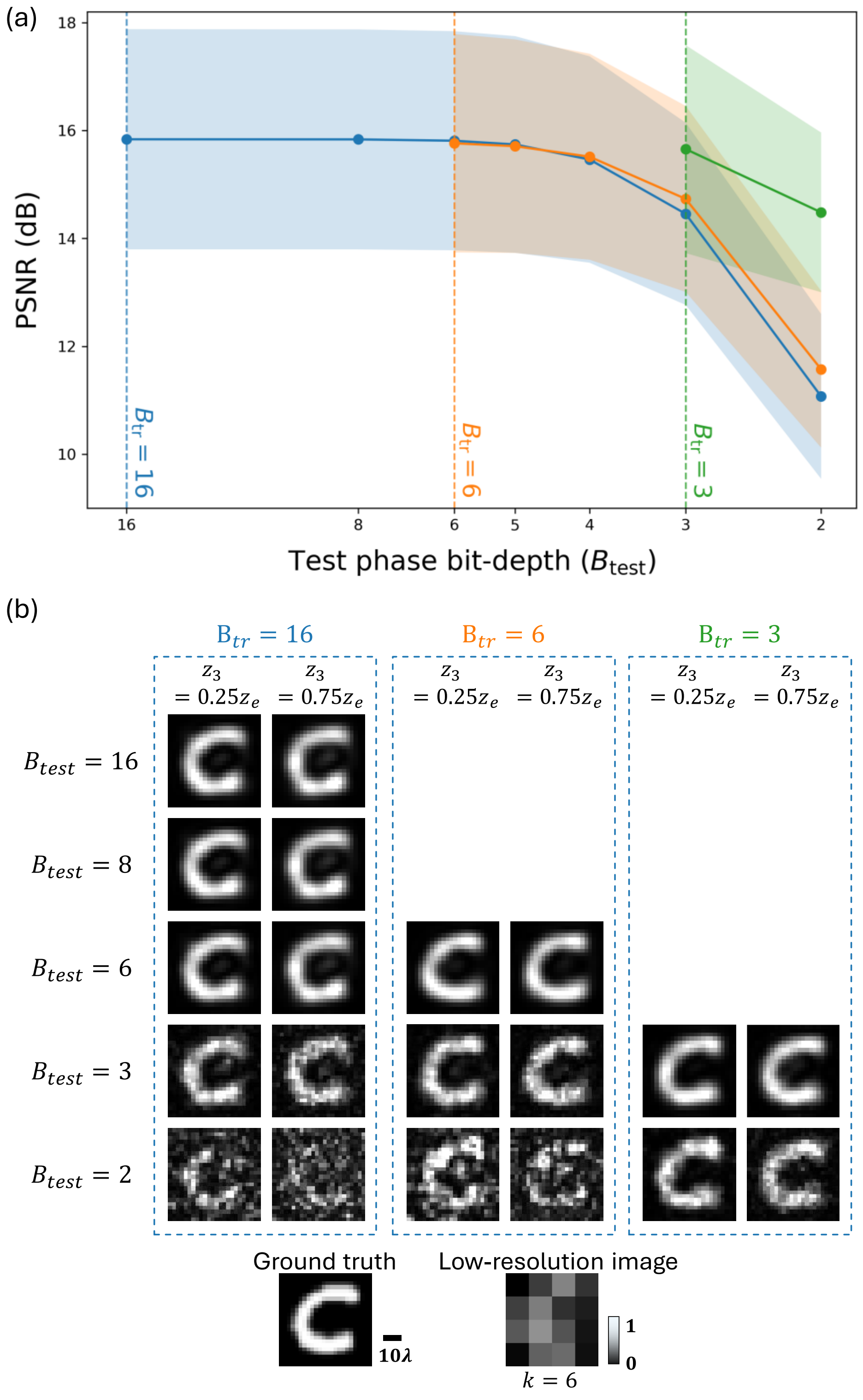

Supplementary Figure S6: Impact of the phase quantization level of the diffractive layers on the EDOF image projection performance of the hybrid system using $k = 6$, $L = 3$, $z_e = 200$ mm and $\lambda = 0.75$ mm. (a) Average PSNR (solid lines) and standard deviation values (shaded regions) are plotted as a function of the test phase bit-depth (B_{test}). The colors correspond to models trained with different quantization

levels (B_{tr}). (b) Projected output images at two axial depths ($0.25z_e$ and $0.75z_e$) under varying testing phase quantization conditions. The ground truth and the low-resolution input images are shown at the bottom for reference.

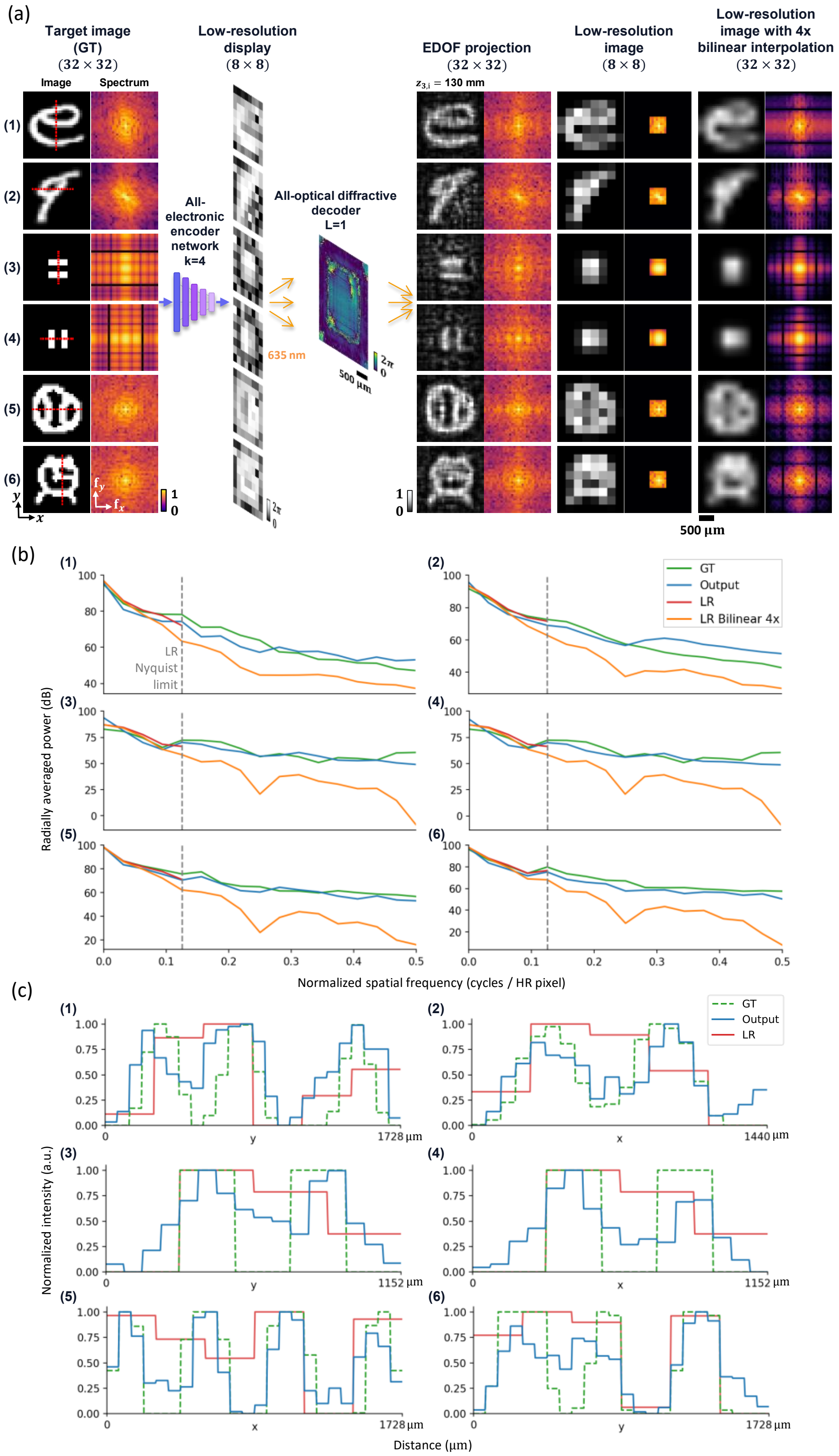

Supplementary Figure S7: Spatial frequency and cross-sectional analysis of the hybrid PSR image projection system using $k = 4$ and $L = 1$. (a) Image projection and 2D spatial frequency spectrum analysis for various test objects, labeled (1) to (6). For each case, we report the ground truth (GT), the EDOF projection image result at

$z_{3,i} = 130$ mm, the low-resolution (LR) image, and the $4\times$ bilinearly interpolated LR image; the images are displayed on the left in each case, and their corresponding 2D spectra are placed on the right. (b) Radially averaged power (dB) as a function of the normalized spatial frequency for the objects shown in (a). The vertical dashed line indicates the LR Nyquist limit. (c) Normalized 1D intensity cross-sections evaluated along the red dashed lines (spanning x and y) marked on the GT images in (a). $\lambda = 635$ nm.

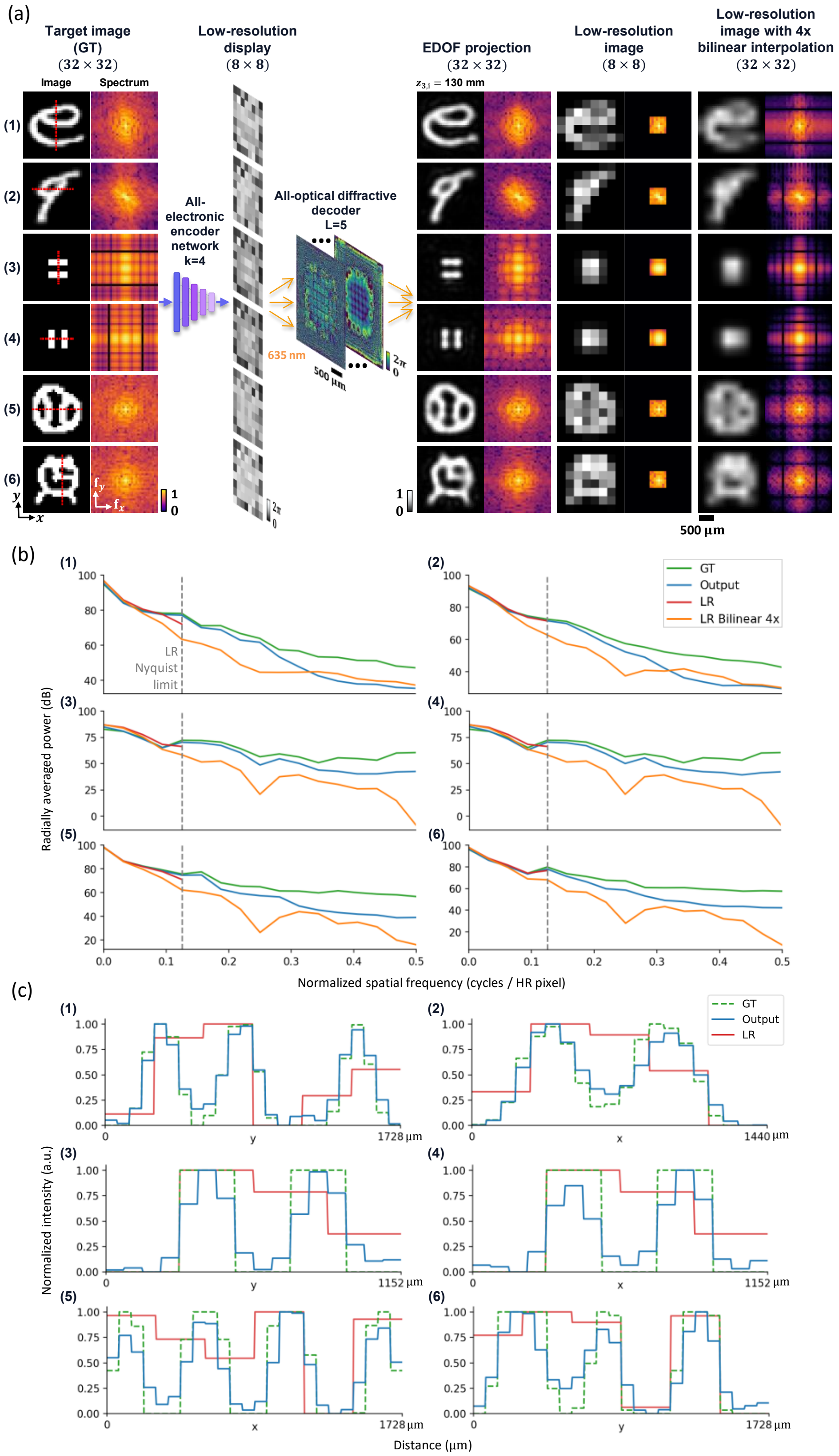

Supplementary Figure S8: Same as in Supplementary Figure S7, except for $L = 5$.
